# Hints for the Sick-Room

**Published:** 1888-07-07

**Authors:** M. M. Basil

**Affiliations:** Author of "The Commoner Accidents to Life and Limb."


### Hints for the Sick-room.

. By M. M. Basil, M.A., M.B., C.M., Author of "The Commoner Accidents to Life and Limb."

### POULTICES, FOMENTATIONS, SINAPISMS, LOTIONS, Etc.

"The simplest way of making charcoal poultices," says one of the text-books, "is to mix one part of charcoal with two parts of linseed meal, and then make the poultice in the ordinary way." Simplicity of all forms, however, does not go hand in hand with efficiency. To secure the deodorising effect of this form of poultices, at least half the quantity of charcoal used, must be brought near the surface of the wound.

A mustard plaster, or sinapism, is not a mustard poultice, and neither a mustard plaster nor a poultice must be allowed to become a mustard blister. A mustard poultice is made by sprinkling some dry mustard on a linseed poultice before its application. It is, however, much better to apply a sinapism first, and a linseed poultice subsequent to its removal.

A sinapism, or mustard plaster, is best made by using ground mustard seeds. It may be applied on rags or tissue paper—not directly, but through fine muslin. Mustard papers are now sold by chemists, and can be applied very quickly; but except in cases where the paper is very thin and pliant they are more painful than ordinary sinapisms. The reason for this difference will be considered a little later on. After removing a sinapism, sprinkle some dusting powder, such as flour, over the area, or moisten it with olive oil, and then protect it with cotton wool. Or, you may remove the mustard plaster and put on a linseed poultice.

The umbilicus, seats of old scars or burns, and the nipples must be avoided in using counter-irritants. You must also be extremely cautious when using mustard in cases of children, especially after fevers (measles), and with patients who are weak, paralysed, or under the influence of stupefying drugs (chloroform, &c.).

What should be the temperature of the water used in making a sinapism? To make it strong, you are recommended by some to use "boiling water" (Domville, Smith, etc.); "vinegar and boiling water," say others (Veitch, Nightingale, etc.); the mildest-mannered ones draw the line at "warm water." Mustard flour already contains the two elements on the chemical reaction of which on each other in presence of moisture its irritant effect depends. The crushing undergone during the process of grinding has prepared everything for the chemical reaction, provided the meal is moistened. Boiling water coagulates one of these bodies, the myronic acid, and prevents the necessary chemical reaction. You may use warm water in cold weather, so as not to chill the patient, but not with the object of making the sinapism more efficient. In the tropics cold water is more grateful to the patient, and the sinapism is long remembered, not so much on the score of the pleasure it gives on its first application, as the furious energy it subsequently displays in its salutary work of torture. Vinegar and other articles only interfere with the reaction, and substitute their own action on the skin, in place of developing that of the mustard flour. As this is the most common example where, to make assurance doubly sure, one is liable to use means which totally defeat the object in view, I shall digress a little to give a caution against other similar mistakes. Condy's fluid is a tolerably good disinfectant, and carbolic acid is a better one; but the natural conclusion that a mixture of the two would be doubly efficient is not valid. Carbolic acid and Condy's fluid in certain proportions nearly destroy each other, and the mixture is perfectly inert. Never, therefore, use them together. Sulphur when burnt in a room is a good disinfectant; chlorine set free by the use of bleaching powder is also very efficient; but they must not be used together, as then they are spent mostly on each other, and the germs of disease go unharmed.

Water-dressings must be well covered and changed two or three times a day; gutta-percha or oil-silk may be used here, as there is no excessive heat to spoil the tissue. Water-dressings are really forms of poultices. Cold lotions, on the other hand, are not meant to be poultices, and must not be covered by any impermeable material.

Turpentine stupes are made by wringing flannels in the same way as in fomentations, and then sprinkling some spirit of turpentine on the fold which is to be placed next the body just before application. The quantity of turpentine required varies in proportion to the surface which is to be reddened; about one to two teaspoonfuls is the average amount. These may be kept on twenty to thirty minutes.

Dry heat can be applied locally with bags containing hot bran, hot salt, or by an oven shelf, hot bricks, stomach-plates, or indiarubber bottles, well covered with flannel; *white* flannel is said to be the best material for this purpose. Other means of securing nearly the same object as that arrived at by the application of dry heat are friction with brandy, slightly moistened with mustard, or with ginger powder.

It is distinctly to be understood that the choice in the external applications described here is never to be made by the nurse herself; a well-trained nurse is not likely to resort to them or to countenance their application merely on the patient's own suggestion. This observation applies to the whole group of these remedies, but especially to hot applications over the abdomen for deep-seated pain. Valuable time may be lost by trifling with a dangerous condition on the ground that the application will do no harm. The application, as a matter of routine, of heat over a mass of bowel already distended with gases under high tension, is not quite free from harm. The pain in such cases is often due to the fact that the parts are unduly stretched by gases in the bowel. Senselessly adding to the force of tension already distending the bowels by rapidly increasing the volume of the gas by heat, is making confusion worse confounded.

Cold may be applied locally by various means. Different forms of indiarubber ice-bags can be easily had now. They should be only half full of ice, and need not be refilled until all the ice has melted. The temperature of a mixture of ice and water, derived from the melted ice, remains at 32 deg. Fahr.—that is, the freezing-point of water—as long as there is the least portion of ice left in the mixture. It rises rapidly after the ice has completely disappeared. As ice is so frequently used in the sick-room in small pieces, it is necessary for you to know the best way to preserve it, and yet have it within easy reach of the patient. Ice melts rapidly if it is allowed to remain in contact with water. The aim, therefore, is to keep it dry, and in contact with bad conductors of heat. A piece of flannel or muslin is thrown across the mouth of a tumbler or jar of convenient size, its centre is depressed somewhat into the tumbler, and the margins securely tied round the rim of the tumbler. You thus form a pocket in which pieces of ice may be kept. The melted portion is filtered out to the lower part of the vessel, and cannot remain in contact with the ice. Where ice-bags are not to be had, gutta-percha tissue may be made into bags of any form by having the apposed margins of them apped-out bag rapidly brushed with chloroform and brought together. These must be tested and found to be waterproof before they are introduced into the sick room. Bladders wrapped in napkins may also be serviceable. It is, however, seldom that one is driven to such shifts.

*(To be continued.)*

